# Cultural adaptation and validation of the Malay Chronic Kidney Disease Self-management instrument (MCKD-SM)

**DOI:** 10.1186/s12882-022-03016-x

**Published:** 2022-12-01

**Authors:** Ayat Ali Al Sawad, Soo Kun Lim, Li Yoong Tang, Aneesa Abdul Rashid, Boon-How Chew

**Affiliations:** 1 Department of Family Medicine, Faculty of Medicine and Health Sciences, Universiti Putra Malaysia (UPM), Selangor, Malaysia; 2 Department of Nursing, King Fahd Hospital of the University (KFHU), Imam Abdulrahman Bin Faisal University (IAU), Dammam, Saudi Arabia; 3 Department of Medicine (Nephrology), Faculty of Medicine, Universiti Malaya (UM), Kuala Lumpur, Malaysia; 4 Department of Nursing Science, Faculty of Medicine, Universiti Malaya (UM), Kuala Lumpur, Malaysia; 5 Clinical Research Unit, Hospital Pengajar Universiti Putra Malaysia (HPUPM Teaching Hospital), Serdang, Malaysia

**Keywords:** Chronic kidney disease, Chronic kidney disease self-management, Malay translation, Nursing care, Pre-dialysis, Self-management

## Abstract

**Background:**

There is growing evidence that self‐management behaviour can improve outcomes for patients with chronic kidney disease (CKD). However, no measures are available in Malay to effectively assess the self-management of CKD. The aim of this study was to translate, culturally adapt and validate the Malay Chronic Kidney Disease Self-Management (MCKD-SM) instrument for Malay-speaking health professionals and patients.

**Methods:**

This study was carried out in two phases: the translation and cultural adaptation phase and the validation phase. The instrument was translated from English to Malay and then adapted and validated in a sample of 337 patients with CKD stages 3–4 attending a nephrology clinic in a tertiary hospital in Malaysia. Structural validity was evaluated by exploratory factor analysis. The instrument’s reliability was assessed by internal consistency and test–retest reliability. The correlations between the MCKD-SM and kidney disease knowledge and the MCKD-SM and self-efficacy were hypothesised a priori and investigated.

**Results:**

The MCKD-SM instrument has 29 items grouped into three factors: ‘Understanding and Managing My CKD’, ‘Seeking Support’ and ‘Adherence to Recommended Regimen’. The three factors accounted for 56.3% of the total variance. Each factor showed acceptable internal reliability, with Cronbach’s α from 0.885 to 0.960. The two-week intra-rater test–retest reliability intraclass correlation coefficient values for all items ranged between 0.938 and 1.000. The MCKD-SM scores significantly correlated with kidney disease knowledge (*r* = 0.366, *p* < 0.01) and self-efficacy (*r* = 0.212, *p* < 0.01).

**Conclusion:**

The MCKD-SM was found to be a valid and reliable patient‐reported outcome measure of pre-dialysis CKD self-management behaviour in the Malay-speaking population.

## Background

Chronic kidney disease (CKD) has emerged as one of the most leading causes of worldwide mortality [1, 2]. The global prevalence of CKD was estimated at 13.4% for all five stages and 10.6% for stages 3–5 [3]. However, the prevalence has been reported in an increasing number of studies and recently estimated to be > 10% of the general population worldwide [4]. Additionally, substantial variation has been found among the Asian population in overall and advanced CKD prevalence (range: 7.0%–34.3% and 0.1%–17.0%, respectively) [5]. In Malaysia, the incidence of CKD is increasing at an alarming rate from 9.07% in 2011 [6] to 15.48% in 2018 [7] due to the growing burden of diabetes, hypertension and the ageing population [7]. Data showed a high number of CKD-related complications, as well as persistent poor disease control and management, particularly among those Malaysian at a late stage of CKD [8]. Studies have shown that progression to advanced stages of CKD results in reduced quality of life and premature death [8]. This progression can be slowed by early disease detection and intervention [9, 10]. However, due to the chronic nature of CKD, patients cannot solely depend on doctors but also need to actively participate in the management of the disease [11]. Hence, to effectively delay the progression of CKD, self-management by patients is an imminent aspect of disease management. According to the literature, patients’ health outcomes improve when they are more active in their own chronic illness treatment [12, 13]. As a result, self-management efforts taken by patients with CKD are integral to controlling the disease’s ongoing symptoms and undesirable sequelae. However, limited studies have been conducted focusing on self-management strategies that address the psychological and behavioural complexities inherent in any chronic disease, as these strategies are vital for effective management of CKD [8]. In addition, studies are lacking on the evaluation of self-management behaviour among patients with early CKD, wherein special attention should be paid to those with low education levels and multiple comorbidities [8]. Given the importance of disease self-management, it is necessary to translate and validate patient-reported outcome measures to facilitate the assessment of self-management among patients diagnosed with CKD in different language contexts.

A viable measurement of self-management behaviour is important for measuring the success of interventions designed to facilitate patients in managing their CKD. However, no patient-reported outcome measures have been identified as suitable for assessing self-management behaviour for the pre-dialysis population [14, 15]. For instance, the Perceived Kidney/Dialysis Self-Management Scale has been deployed to measure perceived self-management behaviour competency among patients with CKD and kidney failure receiving haemodialysis [16]. However, this patient-reported outcome measure was modified from the Perceived Medical-Condition Self-Management Scale template designed to measure self-management behaviour among patients with HIV and diabetes [17, 18].

Individuals who self-manage their chronic conditions actively engage in improving their health, avoiding complications and managing symptoms through adherence to their treatment plans [19]. Therefore, due to the intricate concept of self-management, reliable and valid measures are crucial for capturing accurate empirical data. The CKD Self-Management (CKD-SM) instrument is an example of such a comprehensive measure that can be found in the English language despite its initial development in Chinese and assessment in Taiwan [20]. The CKD-SM can be used to assess how patients self-manage their CKD, to develop more relevant, patient-centred teaching and to implement interventions tailored to the needs of individual patients. This 29-item instrument is composed of four factors. Factor 1: Self-Integration consists of 11 items (7, 11–12, 14, 18–19, 22–23, 25–26 & 28) that examine how a patient attains a balanced life via lifestyle adjustments by incorporating the recommended treatment regimens and self-management activities. Factor 2: Problem-Solving consists of nine items (2, 5–6, 9, 16, 20, 24, 27 & 29) that explore a patient’s capability to seek resources and gain information on CKD to overcome the issues. Factor 3: Seeking Social Support has five items (1, 3–4, 10 & 15) that examine a patient’s capability to seek support from important others to address issues related to CKD. Factor 4: Adherence to Recommended Regimens consists of four items (8, 13, 17 & 21) that assess whether a patient follows the recommended treatment and healthcare regimens. A four-point Likert scale is deployed for all items (1 = never to 4 = always). The total scores for each factor are as follows: Self-Integration (11–44), Problem-Solving (9–36), Seeking Social Support (5–20), and Adherence to Recommended Regimen (4–16). The overall score is between 29 and 116, with higher scores signifying better CKD self-management behaviour. For early-stage CKD, the original English CKD-SM instrument showed good internal consistency, with a 0.88 score for Cronbach’s alpha, and good sampling adequacy, with a 0.89 score for the Kaiser–Meyer–Olkin (KMO) test [20]. A two-week test–retest analysis of the CKD-SM in early-stage CKD indicated good stability with an intraclass correlation coefficient (ICC) of 0.72 [20].

The CKD-SM has been recently translated, culturally adapted and validated in both the Vietnamese and Arabic languages to enable the measurement of self-management behaviour for all CKD stages [14, 15]. Additionally, the reliability and validity of the modified CKD-SM were also assessed in the Australian context [21]. Nevertheless, this instrument needs to be translated and validated into other languages, including Malay, so that the CKD self-management of Malay-speaking countries can be improved. The Malay language refers to the national language of Malaysia and is spoken by the majority of its citizens. Hence, the objective of this study is to translate and culturally adapt the assessment tool from English to Malay and to investigate whether the Malay CKD-SM (MCKD-SM) is a valid and reliable tool in the investigated context.

## Methods

### Design

This study is part of a larger randomised controlled trial to assess the effectiveness of a CKD nurse-led self-management support programme (CKD-NLSM) on kidney disease knowledge, CKD self-management, self-efficacy and quality of life among patients with CKD stages 3–4 [22]. First, forward and backward translations were conducted by a panel of experts who reviewed the translations and conducted cultural adaptation. The MCKD-SM was subjected to psychometric assessment.

### Phase 1: Translation and cultural adaptation process

#### Forward translation and the expert panel

**Fig. 1:**
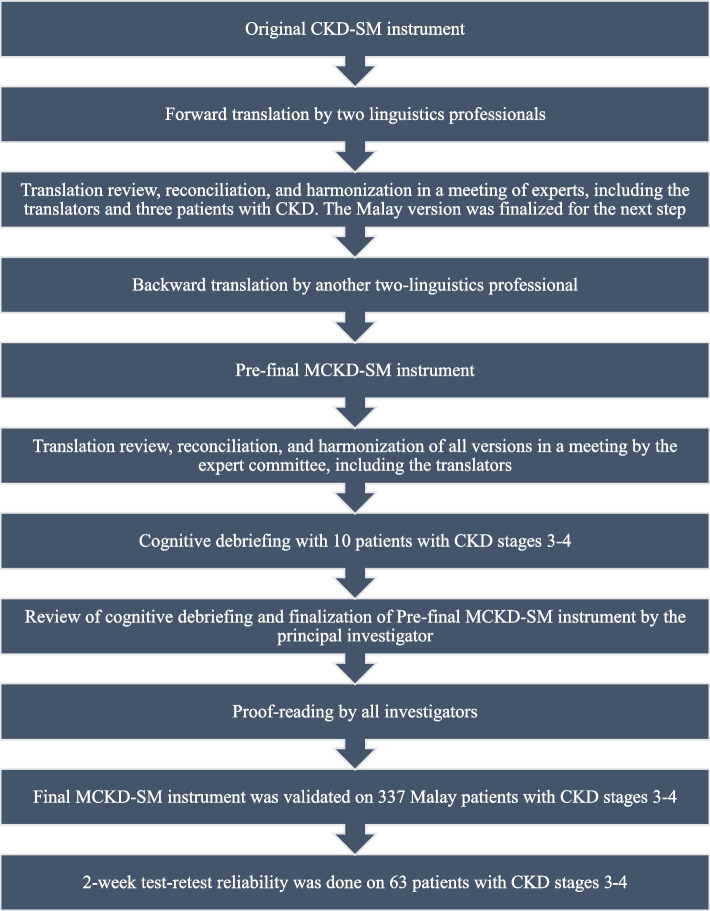
Translation and validation process

Approval to use the CKD-SM was granted by the developer [20]. The English-to-Malay translation process was executed in four steps [23, 24]: forward-translation, expert panel consideration and back-translation, pre-testing and cognitive debriefing, and completion of the final version (see Fig. 1). First, the original instrument was forward translated from English to Malay in an independent manner by two professional bilingual native Malay speakers from the Faculty of Languages and Linguistics, Universiti Malaya. Next, two initial forward translations were discussed, with different points reconciled and harmonized to produce the pre-final MCKD-SM. Next, this pre-final MCKD-SM was assessed in terms of idiomatic, semantic, and conceptual equivalence by 10 experts (1 nephrologist, 2 nursing academicians with experience in instrument validation, 2 CKD nurse-educators, 2 family medicine specialists, and 3 patients with CKD) [25]. Both translations were reviewed by these experts prior to discussions in iteration and the reaching of a consensus on the pre-final MCKD-SM instrument.

#### Back-translation and expert panel

Back translation from Malay to English was carried out by 2 qualified local bilingual translators (with non-medical backgrounds) who were blinded to the original English versions. The translation was re-discussed by the experts to ascertain its similarity to the original instrument, and it was agreed to move the pre-final MCKD-SM to the next stage.

#### Pre-testing and cognitive debriefing

When an instrument is pre-tested, cognitive debriefing is required, in which respondents are asked to verbalize what comes to mind when they hear a question [26]. For this, a sample size of 10–40 individuals is recommended [27, 28]. Therefore, the pre-final MCKD-SM was pilot tested using a purposive sample of 10 Malay-language patients with CKD recruited from the selected nephrology clinic to evaluate the instructions, response format, and the items of the instrument for clarity [25]. The participants filled in the self-administered instrument within 10–15 min. Next, they were requested to provide feedback on the clarity of the instrument’s words and sentences, as well as all aspects of its intelligibility. Subsequently, minor changes were made, such as replacing some translated terms with more commonly used terms, as suggested by the patients. For example, item 2: “Thinking over reasons about bad laboratory data” and item 13: “Don’t follow care providers’ suggestion to exercise” were translated as “Memikirkan sebab-sebab mengenai data makmal yang teruk” and “Tidak mematuhi cadangan penyedia penjagaan untuk bersenam”, respectively. In addition, the patients suggested that ‘teruk’ (bad) be replaced with ‘tidak baik’ (less good), and ‘penyedia penjagaan’ (care providers) be replaced with ‘pengamal perubatan’ (medical practitioners)”.

#### Instrument final version

The final MCKD-SM was then assessed for consistency, and the validation analysis was reflected in the COSMIN (COnsensus-based Standards for the selection of health Measurement INstruments) approach [29].

### Phase 2: Psychometric evaluation

Psychometric measurement properties include structural validity, hypothesis testing for construct validity, internal consistency, and intra-rater test–retest reliability [22]. Exploratory factor analysis (EFA) was performed by applying principal component extraction and Promin rotation methods. In this context, the polychoric correlation method was executed, which is suitable for ordinal variables and items with the Likert-type response scale. Polychoric correlation is advised when the univariate distributions of ordinal items are asymmetric or with excess kurtosis. The Factor 10.10.02 program [30] was applied to conduct the EFA. Parallel analysis was performed to determine the number of factors using the optimal parallel analysis (random permutation) option in the software [31]. A scree plot was used to support the parallel analysis findings.

Internal consistency was examined using Cronbach's α coefficient. Cronbach’s α < 0.70 denotes inadequate consistency, while 0.70–0.90 signifies adequate internal consistency [32]. Intra-rater 2-week test–retest reliability was performed by estimating the ICC. An ICC of 0.70 is the minimum standard for reliability [33]. Similar to previous studies [15, 21], Pearson’s correlation coefficient was used to assess the correlations between kidney disease knowledge using kidney disease knowledge survey (KiKS) [34] and self-efficacy using self-efficacy managing chronic disease (SEMCD) [35] with MCKD-SM.

KiKS measures patients’ knowledge about kidney disease, especially those who do not need to undergo RRT [34]. The 28-item KiKS comprises three factors measuring general knowledge of kidney disease, kidney functions, and progression symptoms. A correct response is given a score of 1; otherwise, 0 is the score. With a total score ranging between 0 and 28, higher scores denote a higher knowledge level about kidney disease.

The 6-item SEMCD measures the self-efficacy of patients with chronic disease [35]. In this instrument, a 10-point Likert scale was employed with scores for answers ranging from 1 (not at all confident) to 10 (totally confident). With the total score ranging from 6–60, higher scores denote better self-efficacy.

It was hypothesised that MCKD-SM would correlate with KiKS and SEMCD at ~ 0.25 [22]. Correlation coefficient scores of 0.20–0.40, 0.40–0.70, 0.70–0.90, and > 0.90 indicate weak, moderate, strong, and very strong correlations, respectively [36].

### Setting

This study was carried out at a nephrology clinic located in a tertiary teaching hospital situated in Kuala Lumpur, Malaysia. The patients there were referred by general practitioners from other healthcare centres. The clinic supports patients with early CKD through those who need renal replacement therapy. Patients with CKD stages 2–5 are managed by nephrologists and qualified CKD nurse-educators.

### Inclusion and exclusion criteria

The following is a list of the inclusion criteria for this study:

- Adults with CKD stages 3–4 (defined as glomerular filtration rate of 15–59 ml/min/173 m.^2^, with evidence of kidney damage)
- Aged ≥ 18 years
- Able to understand, speak and read the Malay language

Additionally, the participants must not have participated in cognitive debriefing and not have been diagnosed with pre-existing cognitive/vision impairment or serious illness (cancer, stroke or dementia).

### Sample size

In light of the psychometric properties analysis, the sample size was based on the 1:10 ratio for each item [25, 33], signifying that 290 participants were needed for this study because the MCKD-SM is composed of 29 items. After considering 20% incomplete responses, 363 (290/0.8) participants were invited to participate in this study. In the two-week intra-rater test–retest testing, at least 50 participants were re-invited to participate [33].

### Data collection and procedure

Data collection was conducted between June 2019 and September 2020. Eligible participants were identified by researchers at the nephrology clinic and recruited using consecutive sampling. After obtaining informed consent from the participants, self-administered instruments on the MCKD-SM, KiKS and SEMCD instrument were distributed to the participants. Demographic data from the participants (gender, age, marital status, ethnicity, employment status, and academic background) were captured in a quiet room located at the clinic. Other medical information, including CKD stages, was gathered from their medical records. Retesting was performed two weeks later.

### Statistical analysis

The gathered data were analysed using the Statistical Package for Social Science (SPSS) version 25.0 (SPSS, Chicago, IL) and FACTOR (10.10.02) software [30]. Descriptive statistics were generated for individual item scores and demographic data. There were no missing data since the principal investigator and research assistants were present to ensure that all participants answered all the items. Any incomplete questionnaires were asked about and clarified by the participants to obtain their responses. The EFA was deployed using principal component analysis, a crucial aspect of tool development, to ensure the content and number of factors in the initial items set. The KMO test and Bartlett’s test of sphericity were also executed.

The retained factors were determined using the following criteria: scree plot, theoretical interpretability of the resulting factor structure and eigenvalues > 1. Next, the items were chosen based on the following four criteria: conceptual coherence of items with individual factor, factor loading > 0.3 [37], no cross-factor loaded items and minimum factor membership of three items. Afterward, the internal consistency of the MCKD-SM was determined based on Cronbach’s α. Test–retest reliability was determined using the ICC with receipt of the completed retest instrument. As for hypotheses testing for construct validity, Pearson correlations among CKD self-management behaviour, kidney disease knowledge and self-efficacy were ascertained, as all scores displayed a normal distribution.

## Results

### Characteristics of the sample

Of the 337 participants who completed the first test, 63 agreed to complete the retest. The participants were between 21 and 87 years old, with a mean *age* of 61.9 (SD = 13.1). Half of them were males (*n* = 192), and 230 were Malay (68.2%). Most of the participants had CKD stages 3a (34.1%, *n* = 115) and 3b (40.9%, *n* = 138). Table 1 lists their characteristics. There were many similarities between patients who participated at baseline (*n* = 337) and those agreed to complete the retest (*n* = 63) in terms of demographic and clinical characteristics.

**Table 1:** Participant characteristics

Characteristics	Mean (SD)	Structural and construct validation (*n* = 337) n (%)	Mean (SD)	Test–retest (*n* = 63) n (%)
Age (year)	61.9 (SD = 13.1)		63.65 (SD = 11.5)	
Gender				
Male		192 (57.0)		36 (57.1)
Female		145 (43.0)		27 (42.9)
Ethnicity				
Malay		230 (68.2)		41 (65.1)
Chinese		39 (11.6)		3 (4.8)
Indian		63 (18.7)		17 (27.0)
Aborigines		1 (0.3)		2 (3.2)
Others		4 (1.2)		0 (0)
Marital status				
Married		249 (73.9)		52 (82.5)
Unmarried		88 (26.1)		11 (17.5)
Education				
Primary level		50 (14.8)		9 (14.2)
Secondary level		163 (48.4)		35 (55.6)
Tertiary level		124 (36.8)		19 (30.2)
Occupation				
Working		93 (27.6)		14 (22.2)
Not working		77 (22.8)		17 (27.0)
Retired		164 (48.7)		31 (49.2)
Student		3 (0.9)		1 (1.6)
CKD stage (GFR mL/min/1.73 m^2^)				
Stage 3a (45‐59)		115 (34.1)		20 (31.7)
Stage 3b (30‐44)		138 (40.9)		31 (49.2)
Stage 4 (15‐29)		84 (24.9)		12 (19.0)

*CKD* chronic kidney disease, *GFR* glomerular filtration rate, *SD* standard deviation

### Structural validity

The EFA was applied to determine the factor structure among the 29 items in the MCKD-SM. Several well-known criteria for factorability of correlation were employed. First, the KMO was 0.909 (> 0.6), and the Bartlett’s test of sphericity was significant (χ^2^_(406)_ = 3735.9, *p* < 0.001). The initial communalities are estimates of the variance in each variable, accounted for by all factors, whereas small values (< 0.3) signify variables that fail to fit the factor solution. Turning to this present study, all initial communalities exceeded the threshold (all loading factors > 0.3) [37].

When compared to the original instrument [20], which has four factors, the results of the parallel analysis on all 29 items identified only three factors: Self-Integration, Seeking Social Support and Adherence to Recommended Regimen. The Problem-Solving factor was absorbed, and the items were distributed into Self-Integration and Seeking Social Support. In addition to the original 11 items of Self-Integration, five items (9, 20, 24, 27 & 29) were moved to Self-Integration from Problem-Solving. Similarly, in addition to the original five items of Seeking Social Support, four items (2, 5, 6 & 16) were moved to Seeking Social Support from Problem-Solving. Hence, the Self-Integration factor of the MCKD-SM had 16 items and was renamed ‘Understanding and Managing My CKD’. Similarly, Seeking Social Support in MCKD-SM had nine items and was renamed ‘Seeking Support’. Four items were retained in the Adherence to Recommended Regimen factor.

**Fig. 2:**
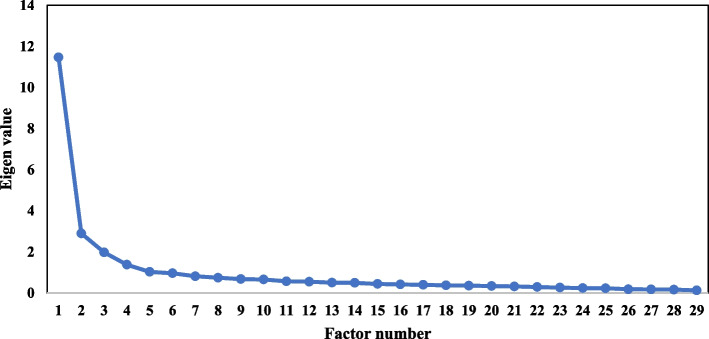
Scree plot for exploratory factor analysis on the Malay Chronic Kidney Disease Self- management (MCKD-SM)

The eigenvalues and total variance explained by the three factors are presented in Table 2 and the scree plot in Fig. 2. The results after Promin rotation revealed that the first factor included 16 items with a loading factor > 0.3. Most items in this factor measured Understanding and Managing My CKD and explained 31.9% of the total variance. The second factor consisted of nine items related to Seeking Support and explained 15.1% of the total variance. The last factor had four items related to Adherence to Recommended Regimen and explained 9.3% of the total variance. The total variance explained by these three factors was 56.3%, which exceeded the recommended value of 50% [38].

**Table 2:** Factor loading and Cronbach’s alpha for the 29-item Malay Chronic Kidney Disease Self-management (MCKD-SM)

	**Factor Loading**		
**Items**	**Factor 1**	**Factor 2**	**Factor 3**
Factor 1: Understanding and Managing my CKD			
26. Adjusting lifestyle to maintain the best condition	0.865		
20. ^a^Actively understanding risk factors of CKD	0.859		
19. Giving up bad habits harmful for kidney	0.857		
22. Managing food portions and choices in social activity	0.840		
12. Heeding habits that may affect kidney function	0.831		
18. Managing CKD to stay healthy	0.824		
25. Managing food followed to care providers’ suggestion	0.800		
29. ^a^Actively seeking information about kidney disease	0.753		
23. Adjusting CKD care to fit new situation	0.721		
14. Changing lifestyle to avoid worse of kidney function	0.705		
11. Merging CKD management into daily life	0.699		
7. Managing food to avoid harm for kidney	0.697		
24. ^a^Finding out reasons for signs and symptoms	0.664		
27. ^a^Utilizing different ways to clarify questions about treatment plan	0.588		
28. Participating selectively in social activities	0.552		
9. ^a^Utilizing different ways to solve problems	0.450		
Factor 2: Seeking Support			
3. Telling family or friends about treatment plan		0.790	
4. Sharing experience with other patients		0.753	
15. Asking family or friends for help when helpless or frustrated		0.748	
1. Discussing with family or friends while questioning or worrying		0.681	
2. ^a^Thinking over reasons about bad laboratory data		0.656	
10. Sharing helpless and frustrated feeling with other patients		0.639	
6. ^a^Finding out possible reasons about high BP value		0.552	
5. ^a^Actively understanding the meaning of laboratory data		0.551	
16. ^a^Actively seeking resources to better control		0.374	
Factor 3: Adherence to Recommended Regimen			
21. Don’t follow care providers’ suggestion to control weigh			0.840
17. Don’t follow care providers’ suggestion to adjust diet habit			0.807
13. Don’t follow care providers’ suggestion to exercise			0.802
8. Don’t follow the dieticians’ suggestion to choose food			0.674
Eigenvalue	9.258	4.376	2.705
% Of Variance	31.9	15.1	9.3
Cronbach α	0.960	0.899	0.885

^a^Originally is Problem-solving; *CKD* chronic kidney disease; Understanding and Managing my CKD originally is Self-integration; Seeking Support originally is Seeking Social Support

### Reliability

The internal consistency of the subscale was very good, with Cronbach’s α ranging from 0.885 to 0.96 (Table 2). The two-week intra-rater test–retest reliability of the MCKD-SM was also very good, with ICC values for all items ranging from 0.938 to 1.000.

### Hypotheses testing for construct validity

A positive link was noted between the MCKD-SM and KiKS scores (*r* = 0.366, *p* < 0.01), which signified that high-level self-management behaviour was related to high-level kidney disease knowledge. Next, the positive link between the MCKD-SM and SEMCD scores (*r* = 0.212, *p* < 0.01) showed that high-level self-efficacy was related to high-level self-management behaviour.

## Discussion

The MCKD‐SM appeared to be a valid and reliable instrument for examining self-management behaviour among Malay‐speaking patients with CKD. The MCKD‐SM successfully measured crucial skills and daily activities for self‐management. The MCKD‐SM was comprehensible to those with poor proficiency. The process of completing the survey took only 10–15 min. The translation and adaptation processes involved in yielding the MCKD-SM adhered to guidelines for the cross-cultural adaptation of self-report measures [23, 24]. Slight variances were noted in linguistic usage, in which the necessary grammatical and cultural amendments were made. Exceptional content validity for the MCKD‐SM was verified by the selected experts with vast experience in caring for patients with CKD and familiar with the medical terms. The inclusion of three patients with CKD with varying backgrounds and work experiences ensured that the instrument’s content was comprehensible, thus increasing the likelihood that the participants would provide accurate responses. Referring to the COnsensus-based Standards for the selection of health Measurement Instruments (COSMIN) checklist, the MCKD-SM displayed acceptable psychometric properties with good structural validity [29]. The COSMIN checklist evaluates the methodological quality of a study on measuring properties of a health-related patient-reported outcome instrument, but does not assess the quality of the health-related patient-reported outcome instrument itself [39]. The checklist was developed with the participation of many experts in the field [39].

Problem-Solving was excluded as a factor in the MCKD-SM, which differs from the original and the Vietnamese, Australian and Arabic versions [14, 15, 21]. This factor (9 items) was dispersed into Understanding and Managing My CKD (originally known as Self-Integration) (5 items) and Seeking Support (originally Seeking Social Support) (4 items). This is related to the local family dynamic, where no individual (health) problem should be kept from the family [40, 41]. Problem-solving in health problems is a family issue and begins with personal understanding of the problem at hand. This is clearly seen in items 20 (‘Actively understanding risk factors of CKD’) and 29 (‘Actively seeking information about kidney disease’), which recorded high scores in Cronbach’s alpha (0.86 and 0.75, respectively) for the Understanding and Managing My CKD factor. The Seeking Social Support factor was renamed Seeking Support due to the inclusion of Problem-Solving items that broadened the support scope to include professional support. This is because patients seek clarification and meaning regarding laboratory results and appropriate actions to better control their diseases [41].

Similar to the case for the original (α = 0.77–0.92) [20] and the Vietnamese (α = 0.77–0.90) [14], Arabic (α = 0.71–0.83) [15] and Australian (α = 0.72–0.85) [21] versions, Cronbach’s α for all factors in the MCKD‐SM were reasonably high and reliable for measuring self‐management behaviour in the Malay‐speaking population. The results of test–retest analyses showed that the MCKD-SM was very stable over the two-week period. In addition, the ICC score was higher than for the original [20] and the Vietnamese [14], Arabic [15] and Australian versions [21]. Nonetheless, the percentage of variance in CKD self-management explained by the MCKD-SM was not high (56.3%). This is ascribed to the participants who were in relatively early CKD stages; thus, many items in the instrument could have appeared less relevant to those with more advanced CKD stages. This is indicated by the < 10% of variance in the Adherence to Recommended Regimen factor, where a healthy diet and exercise might not be immediately relevant to the functioning kidneys at CKD stages 3–4.

In this study, hypotheses testing for construct validity was ascertained by assessing the correlations among kidney disease knowledge, self-efficacy and CKD self-management behaviour. High-level kidney disease knowledge improved CKD self-management behaviour, which is in line with past findings [42, 43]. Better self-efficacy was positively correlated with CKD self-management behaviour, which is in agreement with prior findings [21, 44–46]. Perhaps the weaker-than-expected correlation between CKD self-management behaviour and the SEMCD is because the construct of self-management measured by the MCKD-SM has multiple determinants not limited to kidney disease-specific knowledge and self-efficacy skills [46]. According to Lai [47], age, disease duration, education and depression are other determinants that can affect one’s self-efficacy and self-management in light of pre-dialysis CKD. Younger age, longer disease duration and higher education levels are positively and independently correlated with high-level self-management and self-efficacy. Depression is adversely correlated with self-management and self-efficacy [47]. Similarly, this study outcome revealed that improving generic self-efficacy (SEMCD), such as in symptom control, sufficient social role’s function, healthy emotional functioning and effective communication with doctors, is a potential facilitator of CKD self-management.

This study verifies the reliability, content and structural validity of the MCKD-SM. Essentially, the MCKD-SM should be assessed for its applicability among other patient populations of different societal strata due to the varying cultural, linguistic, healthcare system, healthcare provider and patient expectations, as well as self-management implementation.

### Strengths and limitations

Standard translation, along with rigorous adaptation procedures by a panel of experts, including patients with CKD, imbued the MCKD-SM with exceptional cross-cultural validity. Other strengths include the involvement of participants with a range of ages and CKD stages 3–4 and an EFA-enabling sample size [48]. The correlation with the KiKS and SEMCD supports the potential effects on CKD self-management behaviour from kidney disease knowledge and self-efficacy.

This study has some limitations. First, recruiting from one centre may limit its generalisability to other settings, such as primary care or the lower socioeconomic areas. Second, due to the inclusion criteria, the outcomes are less applicable to Chinese and Indians residing in Malaysia, those who are less proficient in the Malay language or those with CKD stages other than 3 and 4. Hence, future studies should look into validating the MCKD-SM in patient groups of different ethnicities, socioeconomic backgrounds and CKD stages to confirm its validity in measuring CKD self-management behaviour.

## Conclusion

The MCKD-SM is a valid, reliable and feasible patient-reported outcome measure of self-management behaviour in patients with pre-dialysis CKD. This three-factor instrument for measuring CKD self-management behaviour is suitable for clinical practice. Future users should be cautious when comparing the different factors in the MCKD-SM with other versions of the same questionnaire with four factors. However, comparing the questionnaire as a whole (i.e. self-management in patients with CKD) should be fine. Moreover, CKD nurse-educators may deploy it as an assessment instrument when supporting or educating patients during earlier CKD stages, especially regarding understanding and managing CKD, seeking social and professional support, adherence to medications and lifestyle modifications.

## Data Availability

The datasets used and/or analyzed during the current study are available from the corresponding author on reasonable request.

## Abbreviations

CKD: Chronic kidney disease
CKD-SM: Chronic kidney disease self-management
COSMIN: COnsensus-based Standards for the selection of health Measurement Instruments
EFA: Exploratory factor analysis
ICC: Intraclass correlation coefficient
KiKS: Kidney Disease Knowledge Survey
KMO: Kaiser–Meyer–Olkin
MCKD-SM: Malay chronic kidney disease self-management
SEMCD: Self-efficacy for Managing Chronic Disease
